# β-Lactamase Producing *Escherichia coli* Encoding *bla_CTX-M_* and *bla_CMY_* Genes in Chicken Carcasses from Egypt

**DOI:** 10.3390/foods12030598

**Published:** 2023-02-01

**Authors:** Elham Elsayed Abo-Almagd, Rana Fahmi Sabala, Samir Mohammed Abd-Elghany, Charlene R. Jackson, Hazem Ramadan, Kálmán Imre, Adriana Morar, Viorel Herman, Khalid Ibrahim Sallam

**Affiliations:** 1 Oncology Center, Faculty of Medicine, Mansoura University, Mansoura 35516, Egypt; 2 Food Hygiene and Control Department, Faculty of Veterinary Medicine, Mansoura University, Mansoura 35516, Egypt; 3 Bacterial Epidemiology and Antimicrobial Resistance Research Unit, US National Poultry Research Center, USDA-ARS, Athens, GA 30605, USA; 4 Hygiene and Zoonoses Department, Faculty of Veterinary Medicine, Mansoura University, Mansoura 35516, Egypt; 5 Department of Animal Production and Veterinary Public Health, Faculty of Veterinary Medicine, University of Life Sciences “King Mihai I” from Timișoara, 300645 Timișoara, Romania; 6 Department of Infectious Diseases and Preventive Medicine, Faculty of Veterinary Medicine, University of Life Sciences “King Mihai I” from Timişoara, 300645 Timișoara, Romania

**Keywords:** *E. coli*, multidrug resistance, β-lactamase, phylogroups, chicken carcasses

## Abstract

*Escherichia coli* with multidrug resistance and β-lactamase genes may constitute a great public health hazard due to the potential for their transmission to humans through the food chain. This study determined the prevalence, antibiotic resistance profiles, phylogroups, and β-lactamase genes of *E. coli* isolates from chicken carcasses marketed in Mansoura, Egypt. Interestingly, *E. coli* was detected in 98% (98/100) of the chicken carcasses examined, which seemed among the highest contamination rates by *E. coli* worldwide. From the 425 genetically verified *uidA* gene-positive *E*. *coli*, 85 isolates were further studied for antimicrobial resistance profiles, phylogroups, and β-lactamase genes. Interestingly, 89.41% of *E. coli* (76/85) strains tested against 24 different antibiotics were multidrug-resistant. Of the examined 85 *E. coli* isolates, 22 (25.88%) isolates harbored *bla_CTX-M_* and were resistant to ampicillin, cefazoline, and ceftriaxone, while three of them were resistant to ceftazidime besides. Nine (10.59%) *E. coli* strains harbored AmpC- β-lactamase *bla_CMY_* and were resistant to ampicillin. One isolate co-carried *bla_CMY_* and *bla_CTX-M_* genes, though it was negative for the *bla_TEM_* gene. Of the 35 isolates that harbored either extended-spectrum β-lactamase (ESBL) and/or AmpC β-lactamase genes, six strains (17.14%) were assigned to pathogenic phylogroup F and one to phylogroup E, whereas 28 (80%) isolates belonged to commensal phylogenetic groups.

## 1. Introduction

Antimicrobial resistance is one of the universal threats which affect human and animal health especially in developing countries, including Egypt, due to a lack of rules controlling the usage of antimicrobials in different sectors of life (animal, agriculture, as well as human) [1,2]. Food-producing animals usually receive antimicrobials as a treatment or prophylactic courses to protect them from many bacterial infections [3,4]. Such overuse and misuse of antimicrobials results in the production of highly resistant bacteria; even commensal bacteria can acquire such resistance owing to chromosomal mutation or via gaining resistance traits from mobile genetic elements (e.g., plasmids, integrons, and transposons) [5].

The β-lactam antibiotics are one of the most important and widely used antimicrobials for the treatment of bacterial infections in both humans and animals, due to their broad antimicrobial spectrum, good safety profile, and availability of orally used formulations, in addition to their low price in comparison to other antimicrobial categories [6]. They interrupt bacterial cell-wall formation as a result of covalent binding to essential penicillin-binding proteins (PBPs), enzymes that are involved in the terminal steps of peptidoglycan cross-linking in both Gram-negative and Gram-positive bacteria. β-lactam antibiotics are classified into cephalosporins, cephamycins, monobactams, and carbapenems [7].

As a result of the excessive use of β-lactams in animals during the last decades, it is not surprising that acquired resistance to β-lactams has been detected in bacteria of animal and human health concern [8,9,10]. The resistance to β-lactam antibiotics arises as a result of drug inactivation by β-lactamases, an enzyme produced by bacteria that hydrolyze β-lactam antibiotics [11].

Extended-spectrum β-lactamases (ESBL)-producing bacteria result from mutations in the *bla_TEM-1_* and *bla_SHV-1_* β-lactamase genes with the global dissemination of ESBL-encoded by *bla_CTX-M_*-producing organisms [12] exhibiting resistance to penicillin, oxyiminocephalosporins, and monobactams. The ESBL-producing bacteria cannot hydrolyze cephamycins and are suppressed by clavulanic acid [13]. Meanwhile, ampicillin class C β-lactamase (AmpC) enzymes are active on cephamycins, oxyiminocephalosporins, and monobactams [13]. The genes encoding the expanded spectrum ß-lactamases are mostly located on mobile genetic elements (e.g., plasmids or transposons), which enable them to be easily mobilized and transferred by horizontal gene transfer from one bacterium to another, even between different bacterial species [5], resulting in frequent treatment failures or reduced efficacy of the therapeutic agents when employing broad-spectrum β-lactams as one of the first lines of treatments in human medicine. Egypt has been listed among the countries with a high prevalence of extended-spectrum β-lactamases (ESBL), and AmpC b-lactamases resistance, in various Gram-negative bacteria from animals and humans [14,15]. 

*Escherichia coli* are among the most important foodborne pathogens that contaminated poultry meat and implicated worldwide in public health concerns [16]. Superbug *E. coli* strains have been disseminated all over all kinds of samples (environment, food, water, and human). Numerous studies revealed that antimicrobial-resistant *E*. *coli* infections in humans were caused by strains of animal origin [17,18,19]. β-lactamases-producing *E. coli* organisms have increased worldwide over the last decades irrespective of whether the bacteria are commensal or pathogenic [20,21]. The issue about the existence of such resistance genes in organisms is that such resistance genes are plasmid-mediated and can be transferred easily from one bacterium to another [22]. This may explain the implication of *E. coli* in the transmission of resistance genes to humans through food sources [23,24].

In Egypt, due to the continuous administration of antimicrobials of β-lactams classes at subtherapeutic doses for growth promotion and as prophylactic measures in the poultry industry sector, ESBL and AmpC β-lactamase-producing Gram-negative bacteria, especially *E. coli,* have frequently been isolated from poultry clinical samples [25,26]. Therefore, this study was conducted to screen poultry meat for its contamination with such resistant strains, which is considered a critical source for public health, via the food chain, resulting in foodborne infection of strict treatment options, highlighting the need to monitor the use of such antibiotics, as well as to establish continuous epidemiological study for the rapid detection of such resistance via human food chain sources.

## 2. Materials and Methods

### 2.1. Sample Collection and Preparation

A total of 100 whole local frozen chicken carcasses were randomly collected from 25 retail shops and supermarkets distributed in Mansoura city, Egypt, during the period of December 2016 to May 2017, for isolation and molecular characterization of *Escherichia coli*. Each individual sample was aseptically packaged into a clean polyethylene bag, marked, and transferred in an icebox to the Laboratory of Food Hygiene and Control Department, Faculty of Veterinary Medicine, Mansoura University, wherein the microbiological analysis was performed. 

### 2.2. Isolation and Identification of Escherichia coli

Each whole poultry carcass was separately rinsed with 225 mL of sterile tryptone soya broth (CM0989; Oxoid, Hampshire, UK) and the suspension obtained was incubated at 37 °C, for 18–24 h. Enriched cultures were plated onto sterile MacConkey agar (CM1169; Oxoid, Hampshire, UK), then the plates were incubated for 24 h at 37 °C. Up to five typical presumptive *E. coli* colonies were sub-cultured onto nutrient agar slopes and incubated for 24 h at 37 °C for further biochemical and molecular identification. A total of 600 typical colonies were subjected to various biochemical tests for further identification and confirmation of *E. coli*.

### 2.3. Molecular Identification of E. coli

For molecular confirmation, the biochemically positive *E*. *coli* isolates (*n* = 434) were subjected to PCR for amplification of the *uidA* gene, the gene-specific for pathogenic *E coli* [27]. For chromosomal DNA isolation, 5 mL from the bacterial culture in nutrient broth (CM0001; Oxoid, Hampshire, UK) was incubated overnight at 37 °C, followed by centrifugation at 3000 rpm for 15 min. The supernatant was removed and the pellet was re-suspended in 1 mL nuclease-free water, homogenized, transferred to a 1.5-mL Eppendorf tube, vortexed, and placed in a heat block at 70 °C for 15 min. The tubes containing the heated lysate were then centrifuged and the resultant supernatant was used as a DNA template.

*Escherichia coli* strains isolated from examined chicken carcasses were screened for the presence of the *uidA* gene using a primer set for the amplification of the *uidA* gene with the following sequence 5′-ATGCCAGTCCAGCGTTTTTGC-3′ and 5′-AAAGTGTGGGTCAATAATCAGGAAGTG-3′ for sense and antisense, respectively. The primer set can amplify a DNA of 1487 bp (Eurofins Genomics^®^, St. Charles, MO, USA). PCR amplification was carried out in a 20-µL reaction mixture using a ready-to-use solution of GoTaq Green Master Mix, (Promega Corporation^®^, Madison, WI, USA) supplied in 2× Green GoTaq reaction buffer (pH 8.5). PCR reaction mixture consisted of 10 µL GoTaq Master Mix 2×, 1 µL (10 pmol) from each of sense and antisense primers, 2 µL DNA template, and 6 µL autoclaved water. The mixture was subjected to 30 cycles of amplification in a Gene Amp PCR system 2700 (Applied Biosystems^®^, Foster City, CA, USA). The first cycle was preceded by denaturation at 95 °C for 5 min. Each cycle performed for the *uidA* gene consisted of denaturation for 45 s at 95 °C, annealing for 45 s at 63 °C, and extension at 72 °C for 1 min, followed by a final extension at 72 °C for 5 min by the end of the last cycle, then cooling at 4 °C. The PCR products were separated through running agarose gel (1.5% agarose) electrophoresis after loading 5 μL of the PCR mixture for 35 min at 100 V. The amplified DNA was then stained in an ethidium bromide solution (Sigma-Aldrich^®^ Co., St. Louis, MO, USA), followed by visualization and photographing under ultraviolet light.

### 2.4. Antimicrobial Susceptibility Testing 

The susceptibility testing of eighty-five selected *E*. *coli* isolates against 24 chosen antimicrobials (ampicillin, piperacillin, ampicillin/sulbactam, piperacillin/tazobactam, ticarcillin/clavulanic, aztreonam, cefazolin, ceftriaxone, ceftazidime, cefepime, levofloxacin, gentamicin, tetracycline, tobramycin, trimethoprim/sulphamethoxazole, minocycline, amikacin, ertapenem, meropenem, doripenem, imipenem, nitrofurantoin, ciprofloxacin, and tigecycline) was carried out by determining the minimum inhibitory concentration (MIC) using the Sensititre semi-automated susceptibility system (Trek Diagnostic Systems^®^, Inc., Westlake, OH, USA) and the Sensititre Gram-negative plate GN4F according to the manufacturer’s directions. The results were interpreted according to the Clinical and Laboratory Standards Institute (CLSI) [28], except for tigecycline, which was tested according to European Committee on Antimicrobial Susceptibility Testing (EUCAST) breakpoints [29].

*Escherichia coli* ATCC 25922, *Pseudomonas aeruginosa* ATCC 27853, *Enterococcus faecalis* ATCC 29212, and *Staphylococcus aureus* ATCC 29213 were used as the control for the determination of MIC. 

### 2.5. PCR Screening for β-Lactamase Genes

The confirmed *Escherichia coli* strains isolated from examined chicken carcasses that showed resistance to beta-lactam antibiotics were screened for β-lactam resistance genes (*bla_TEM_*, *bla_CTX-M_*, *bla_SHV_*, *bla_OXA_*, *bla_CMY_* genes) using PCR amplification [30,31,32,33] The details concerning primer sequences, annealing temperatures, and amplified product sizes are summarized in Table 1.

### 2.6. Phylogenetic Typing of E. coli Isolates

The confirmed *E. coli* strains isolated from chicken carcasses were examined using the quadruplex phylotyping PCR method according to Clermont et al. [34] based on the quadruplex genotype to detect the presence/absence of the four genes (*arpA*, *chuA*, *yjaA*, and *TspE4C2*). *E. coli* ATCC 25922 and ATCC BAA-196 served as control positive strains. Quadruplex PCR was conducted using GoTaq Green Master Mix (Promega Corporation^®^, Madison, WI, USA). A reaction mixture of 20-µL volume consisted of 10 µL GoTaq Green Master Mix, 2 µL of DNA template, and 1 µL (20 pmol) for each of the sense and antisense primers of the four genes. The PCR was performed using a Perkin-Elmer GeneAmp 9600 Thermal Cycler in which the mixture was subjected to 30 cycles of amplification. The annealing temperatures for the different amplified genes were described in Table 1. The electrophoresis of PCR products was mentioned before. 

## 3. Results and Discussion

### 3.1. Prevalence of E. coli in the Chicken Samples Examined

**Figure 1:**
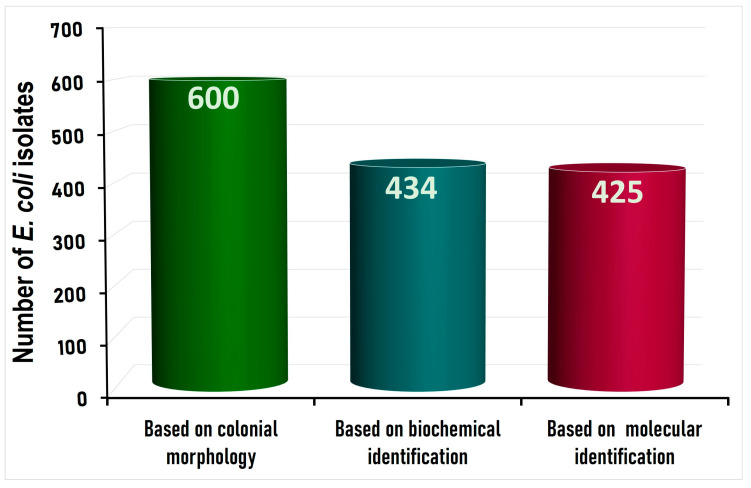
Number of *E. coli* isolates identified depending on colonial morphology, biochemical and molecular identification.

*Escherichia coli* was molecularly detected at a very high prevalence rate of 98% (98/100) among the 100 chicken carcasses tested. A total of 600 colonies (circular bright, pink-colored colonies) were selected from the 100 chicken carcasses, based on their morphological identification. Among these 600 colonies, 434 isolates were confirmed biochemically (production of indole from tryptophan, Methyl red test, Voges–Proskauer test, Citrate utilization test, Urease test) as *E. coli* and were further subjected to molecular confirmation by PCR for detection of the *uidA* gene: the *E. coli* marker gene. Interestingly, 97.92% (425/434) of the biochemically identified isolates were positive for the *uidA* gene (Figure 1), indicating that the biochemical tests can be used as an accurate tool for the identification of *E*. *coli*. A supplementary figure for the amplified DNA product of *uidA* gene for molecular verification of *E*. *coli* is presented in Figure S1.

The *uidA* gene is very specific to *E. coli*, especially the pathogenic strains associated with foodborne infection outbreaks, and is frequently identified by the redundant detection of *uidA* by PCR. The occurrence of *E. coli* at a high percentage in 98% of poultry carcass samples indicates poor hygienic measures during poultry carcass processing.

In developing countries, including Egypt, the use of conventional methods of slaughtering and evisceration resulted in poultry exposure to high contamination levels with different types of bacteria such as *E. coli*, which is considered a normal inhabitant of the intestinal tract of the live birds [36]. The present study is considered among those which showed the highest prevalence rate of *E*. *coli* in poultry carcasses. Likewise, high *E. coli* prevalence rates of 93.7% [37] and 90% [38] were reported in raw poultry carcasses in Egypt, and also by rates of 89% in Qatar [39], 87.5% in Turkey [40], and 60% in Bangladesh [41]. Such a high prevalence of *E. coli* could result in the production of inferior quality poultry meat with subsequent economic losses and public health hazards of great concern. 

On the contrary, lower contamination rates of poultry meat by *E. coli* organisms had been recorded in former studies performed Egypt with an incidence of 37.7% [42], 35% [43], and 11.7% [25]. Lower *E. coli* contamination rates in poultry meat were also recorded worldwide. For instance, prevalence rates of 39.76%, 38.7%, and 40.82% were reported in India [44], the USA [45], and Bangladesh [46], respectively. The variations in the *E. coli* contamination rates among different studies depends on the hygienic facilities available through the poultry processing system. Therefore, strict hygienic measures during poultry carcass processing should be adopted.

### 3.2. Antimicrobial Resistance Profiles of E. coli Isolates

**Figure 2:**
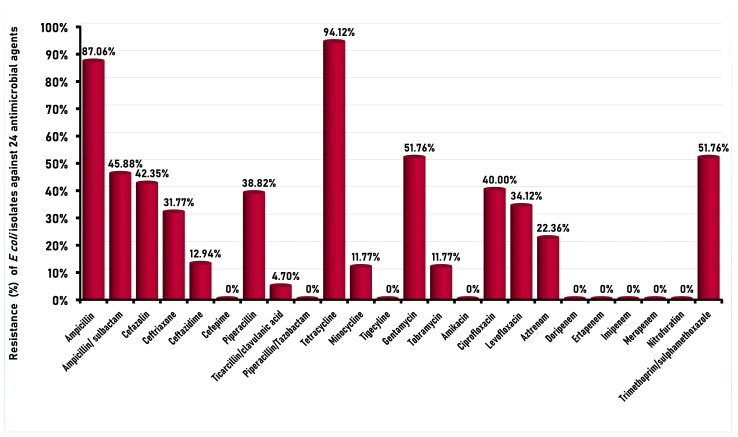
Antimicrobial resistance profiles of *E. coli* derived from poultry carcasses (*n* = 85) tested against 24 antibiotics in Egypt: ampicillin (R ≥ 32 μg/mL), ampicillin/sulbactam (R ≥ 32/16 μg/mL), doripenem (R ≥ 4 μg/mL), ticarcillin/clavulanic (R ≥ 128/2 μg/mL), aztreonam (R ≥ 16 μg/mL), cefazolin (R ≥ 32 μg/mL), ceftriaxone (R ≥ 4 μg/mL), ceftazidime (R ≥ 16 μg/mL), cefepime (R ≥ 16 μg/mL), ciprofloxacin (R ≥ 4 μg/mL), levofloxacin (R ≥ 8 μg/mL), piperacillin (R ≥ 128 μg/mL), gentamicin (R ≥ 16 μg/mL), tetracycline (R ≥ 16 μg/mL), tobramycin (R ≥ 16 μg/mL), amikacin (R ≥ 64 μg/mL), piperacillin/tazobactam (R ≥ 128/4 μg/mL), ertapenem (R ≥ 2 μg/mL), trimethoprim/sulphamethoxazole (R ≥ 4/76 μg/mL), imipenem (R ≥ 4 μg/mL), minocycline (R ≥ 16 μg/mL), meropenem (R ≥ 4 μg/mL), nitrofurantoin (R ≥ 128-μg/mL), tigecycline (R ≥ 2 μg/mL). The minimum inhibitory concentration (MIC) breakpoints were carried out according to the CLSI [28] and EUCAST [29] guidelines; R: resistant.

*E. coli* isolates from chicken carcasses exhibited variable degrees of resistance against the 24 antimicrobials tested (Figure 2). The highest resistance rate by more than 94% of the isolates was demonstrated against tetracycline, followed by a resistance rate of 87.06% of the isolates to ampicillin. To a lesser extent, resistance rates of isolates to trimethoprim/sulfamethoxazole, gentamicin, ampicillin/sulbactam, and cefazolin reached 51.76%, 51.76%, 45.88%, and 42.35%, respectively. On the other hand, all the isolates were susceptible to amikacin, cefepime, doripenem, piperacillin/tazobactam, ertapenem, imipenem, meropenem, nitrofurantoin, and tigecycline. Of the 85 *E. coli* strains tested, only one strain showed susceptibility to all antibiotics used in the study. 

In the current study, the higher resistances rates of the *E. coli* strains isolated from poultry meat against tetracycline and ampicillin were in concordance with previous studies conducted in Egypt; in which 94.7% and 100% [47], as well as 80.9% and 71.4% [25], of *E. coli* isolates recovered from poultry meat were resistant to tetracycline and ampicillin, respectively. A similar pattern of antibiotic resistance of *E. coli* strains isolated from poultry meat had been recorded in previous studies in other countries [48,49], indicating the selective pressure of these antimicrobials in the treatment of *E. coli* infections in poultry due to the rational use of antimicrobial drugs in poultry production for treatment, subtherapeutic or prophylactic purposes, in addition to increasing productivity [3,50,51]. Therefore, a strict system should be established to control the use of antimicrobials not only for the animal, but also in the agricultural sectors.

Among the 85 *E. coli* isolates examined, 76 isolates (89.41%) were resistant to antimicrobials of three different classes or more, making them categorized as multidrug-resistant strains. Multidrug resistance (MDR) among *E. coli* strains isolated from poultry in previous studies exhibited a high prevalence of 69.1% [52] and 83% [53] of the *E. coli* isolates. The high prevalence of resistance patterns against three or more classes of antimicrobials could be related to the different antibiotic regimens used for the different livestock species [54,55]. In fact, it is nowadays accepted that the overuse of antibiotics in animals and poultry is the main driver for the dissemination of multi-drug resistance [56]. It is estimated that there is more extensive antibiotic use in livestock and poultry than in human medicine. The use of antimicrobials in animal production is associated with the emergence and spread of AMR in food-related bacteria and leads to the selection of antimicrobial resistance among pathogenic and commensal bacteria in the intestinal tract of food animals. Therefore, resistant bacteria can contaminate food products and colonize the human microbiota via the food chain by the handling and/or consumption of contaminated foods [57,58].

The present study indicated extensive contamination of chicken carcasses examined with multidrug-resistant *E. coli* that may constitute a tremendous threat to public health. The competent authorities should therefore enforce antimicrobial resistance (AMR) regulation to confirm the cautious use of antimicrobials to reduce the risk of transmission of antimicrobial-resistant organisms via the food chain. Farmers should be prevented from the unsystematic use of antimicrobials in poultry production and promoted to implement preventive measures by observing biosecurity in addition to good management practices. 

Extended-spectrum β-lactamase (ESBL) and AmpC β-lactamase *E. coli* exhibited resistance against third-generation cephalosporins (ceftriaxone and ceftazidime) antibiotics detected in 35 (41.18%) *E. coli* isolates recovered from poultry meat in this study. Such prevalence of ESBL-producing *E. coli* is consistent with the prevalence rates reported in previous studies on ESBL *E. coli* in chicken meat in which ESBL-producing *E. coli* was recorded in Ghana with a prevalence rate of 52.8% [59], as well as in Germany, with a rate of 38.7% [60]. On the contrary to the findings of the current study, a lower prevalence of ESBL had been isolated from poultry meat in Tanzania at a rate of 20.1% [61] and in Pakistan by rates of 7.76% [62]. It has been indicated that among 52.9% AmpC-producing Enterobacteria, most isolates were identified as *Escherichia coli* [60]. It is noteworthy that many publications found that extended-spectrum β-lactamase (ESBL) and AmpC-producing *E. coli* present in humans and animals mostly shared identical sequence types (STs), suggesting the transmission of such resistant genes and human infections with ESBL-producing *E. coli* of animal source [63].

### 3.3. Determination of β-Lactamase Genes

The β-lactamase encoding gene *bla_TEM_* conferring resistance to penicillins was detected in 67.06% (57/85) of the *E. coli* isolated from the poultry carcasses. Twenty-six (25.88%) of the *E. coli* strains harbored *bla_CTX-M_* and were resistant to ampicillin, cefazoline, and ceftriaxone, while three of them were resistant to ceftazidime as well (Table 2). Ten (11.76%) *E. coli* strains harbored AmpC-β-lactamase *bla_CMY_* and were resistant to ampicillin. Six of them exhibited resistance to cefazoline and ceftriaxone harboring *bla_TEM_*, while two among these ten strains were also resistant to ceftazidime (Table 3). Six strains of the *bla_CMY_* and the *bla_CTX-M_* harboring *E. coli* strains were holding *bla_TEM_* genes (Table 2 and Table 3). One strain was holding both *bla_CMY_* and *bla_CTX-M_* genes and was negative for *bla_TEM_*. No *E. coli* strains were positive for *bla_SHV_* and *bla_OXA_*_._ The PCR-amplified DNA products of each of *bla_TEM_*, *bla_CTX-M_*, and *bla_CMY_* genes are presented in a supplementary figure (Figure S2).

It is well known that among the β-lactamase genes, *bla_TEM_* is the most predominant one that is widely spread in Gram-negative bacteria, encoding enzymes that hydrolyze penicillin and first-generation cephalosporins. In accordance with our findings concerning the predominance (67.06%) of *bla_TEM_*, previous studies confirmed such predominancy with prevalence rates of 92% [64] and 80% [65] in *E. coli* isolated from poultry. On the contrary, *bla_CTX-M_* was the predominant (58.1%) ESBL gene detected in *E. coli* recovered from chicken meat in the Netherlands [66].

Several studies in Egypt confirmed the spreading of *bla_CTX-M_* in different sources including food [14,25]. The *bla_CTX-M_* gene was formerly identified in ESBL-producing *E. coli* isolates from chicken and beef samples examined in Egypt [52]. Likewise, five ESBL *E. coli* strains harboring *bla_CTX-M_* were isolated from raw and ready-to-eat beef products [67], with the same *bla_CTX-M_* gene prevalence recorded in the present study. Globally, *bla_CTX-M_* was revealed as the predominant ESBL gene in ESBL-producing isolates [21], as major public health importance pathogen [68].

The AmpC β-lactamase *bla_CMY_* had been previously detected in six *E. coli* strains isolated from colibacillosis-infected chickens in Egypt [69], although this gene could not be detected in *E. coli* recovered from sound poultry meat [25]. It has been demonstrated that the first isolation of *E. coli* strains encoding the AmpC-resistant gene *bla_CMY_* in Egypt was recovered from hospitalized patients with urinary tract infections in three Egyptian hospitals [70]. Our study is the first that recovered *E. coli* strains harboring the *bla_CMY_* gene from sound (non-infected) chicken meat in Egypt.

All the ESBL and AmpC-producing *E. coli* strains isolated from poultry carcasses in the current study showed resistance against further antibiotics of different antimicrobial classes other than β-lactam. In this context, previous studies indicated that ESBL- and AmpC-producing bacteria are frequently cross-resistant to other antimicrobials such as aminoglycosides, chloramphenicol, tetracyclines, sulfonamides, trimethoprim, or quinolones, usually due to the existence of different resistance genes on mobile genetic elements such as plasmids, transposons, or integrons [71,72,73].

### 3.4. Phylogroup Characterization of Isolated E. coli Strains

**Figure 3:**
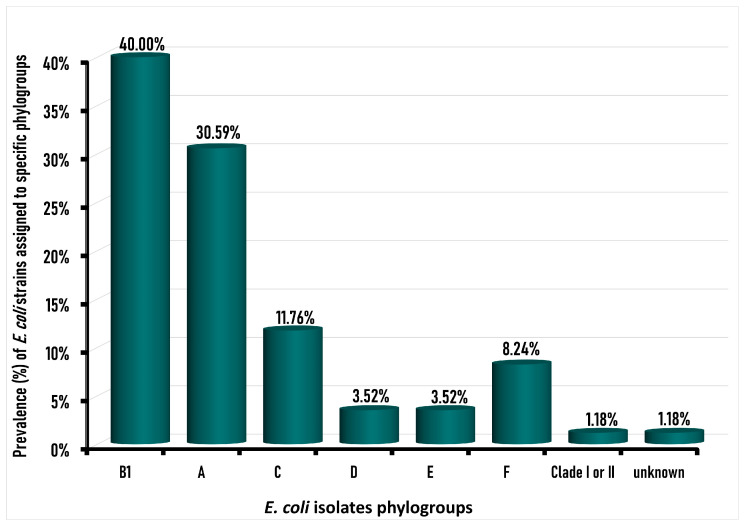
Prevalence of different phylogroups among total *Escherichia coli* isolates (*n* = 85).

The frequency distributions of different *E. coli* phylogroups among the tested *E. coli* isolates from chicken carcasses are illustrated in Figure 3.

Among the 85 *E. coli* isolates, the majority (70/85; 82.35%) were identified as commensal strains with a phylogroup of B1, A, or C, divided into phylogroup B1 (34/85; 40%), followed by phylogroup A (26/85; 30.59%), and phylogroup C (10/85; 11.76%). The pathogenic phylogroup *E. coli* was detected in 13 (15.29%) of the 85 *E. coli* isolates, which were distributed as 7 (8.24%) phylogroup F, 3 (3.52%) for each of D and E phylogroups, and only 1% was Clade I or II, with an absence of phylotype B2. The amplification of the genes incorporated in the phylogroup typing of the confirmed *E*. *coli* strains is presented as a supplementary figure (Figure S3) showing a representative agarose gel electrophoresis of the DNA for the *arp*A, *chu*A, *yja*A, and *trp*A (quadruplex PCR), along with TspE4C2, *trp*A (group C) (singleplex), and *arp*A (group E).

Similar to the current study, the predominant phylogroup among the *E. coli* strains detected in previous studies in retail chicken samples worldwide were phylogroup B1 followed by phylogroup A, such as in Brazil [74,75], Italy [76], Ghana [51], and Pakistan [77], as well as in Egypt in poultry in which phylogroup B1 was the predominant phylogroup among the *E. coli* isolates (74%), followed by phylogroup C (20%), and then pathogenic phylogroup D (4%), while there were no *E. coli* strains possessing phylotypes A, B2, E and F [26].

Of the 35 *E. coli* which exhibited resistance against third-generation cephalosporin (ceftriaxone and ceftazidime) antibiotics in this study, six (17.14%) strains belonged to pathogenic phylogroup F and one to phylogroup E. However, the other 28 (80%) were considered as commensal *E. coli* strains belonging to commensal phylogenetic groups. Concerning our *E. coli* isolates that harbor *bla_CTX-M_*, 22.72% were phylogroup A, 50% belonged to phylogroup B1, while 13.63% belonged to each of phylogroup C and phylogroup F (Table 4). Similarly, 22.22% of *bla_CMY_*-positive *Escherichia coli* isolates were phylogroup A, while 33.33% were categorized as phylogroup B1 and F, whereas 11.11% only belonged to phylogroup C (Table 4).

It has been confirmed that the predominant phylogenetic grouping of extended-spectrum β-lactamases and AmpC-producing *E. coli*, which exhibited resistance against third-generation cephalosporins and were isolated from broiler chickens, were mainly type B1 and A (commensal phylogroup), followed by type D and B2 (pathogenic phylogroup) [60,78]. However, all (100%) ESBL *E. coli* isolated from diseased poultry samples in a previous study conducted in Egypt, belonged to phylogroup D, while most (66%) of the AmpC strains belonged to phylogroup B1 [69], which is quite opposite to that reported in our study.

The results of the current study might lead to the assumption that resistant group F isolates from chicken carcasses may contribute to the dissemination of antimicrobial resistance in humans, resulting in limited treatment of *E*. *coli* infection. It has been reported that *E. coli* belonging to phylogroup F that were isolated from chicken samples were closely related to the extraintestinal pathogenic *E. coli* causing human infections [79]. 

Above and beyond the fact that urinary tract infections (UTIs) are among the most common infections both in community and hospitals, they are most frequently caused by multidrug-resistant *E. coli* that challenge UTI treatment. Although the wide spreading of the phylogroups B2, F, and D pathogenic *E. coli* strains cause extraintestinal infection, the commensal *E. coli* strains belonging to the most newly diverged phylogroups are also responsible for severe intestinal infections. 

## 4. Conclusions

The present study reported high contamination rates of chicken carcasses with multidrug-resistant *E. coli* contaminants, along with the isolation of pathogenic *E. coli* isolates harboring extended-spectrum β-lactamases and AmpC-β-lactamases-encoding genes *bla_CTX-M_* and *bla_CMY_*. To our knowledge, this is the first study isolating *bla_CMY_* from sound chicken meat for human consumption in Egypt. The occurrence of *E*. *coli* in almost all of chicken samples indicates poor hygiene and highlights the antimicrobial resistance problem in Egypt caused by the rational use of antimicrobials in animal husbandry and calls for a nationwide surveillance program to monitor antimicrobial resistance. These findings provide evidence that healthy broilers in Egypt could be a source for the dissemination of transmissible resistance mechanisms among foodborne pathogens brought from the unhygienic environment of the food chain during slaughtering. Although the majority of the isolated strains were commensal *E*. *coli*, about 16% of the strains were pathogenic, while 7% were pathogenic and categorized as a phylogroup F that harbored plasmid-mediated antibiotic-resistant genes, which may lead to infections via consumption of contaminated food with no treatment in humans. In addition, the commensal *E*. *coli* strains which harbor antibiotic-resistance genes, which are mainly plasmid-mediated strains, can be easily transmitted to further pathogenic bacteria that may lead to human infection with no treatment.

## Figures and Tables

**Table 1:** Primers used for PCR amplification and DNA sequencing.

Target Gene	Primer Sequence	PCR Product Size	Annealing Temperature (°C)	Reference
*bla_CTX-M_*	F: 5′-CGCTTTGCGATGTGCAG-3′ R: 5′-ACCGCGATATCGTTGGT-3′	550 bp	55	[30]
*bla_SHV_*	F: 5′-AGGATTGACTGCCTTTTTG-3′ R: 5′-ATTTGCTGATTTCGCTCG-3′	795 bp	64	[31]
*bla_OXA_*	F: 5′-TATCTACAGCAGCGCCAGTG-3′ R: 5′-CGCATCAAATGCCATAAGTG-3′	591 bp	61	[32]
*bla_TEM_*	F: 5′-ATAAAATTCTTGAAGACGAAA-3′ R: 5′-GACAGTTACCAATGCTTAATC-3′	1080bp	51	[30]
*bla_CMY_*	F: 5′-GACAGCCTCTTTCTCCACA-3′ R: 5′-TGGAACGAAGGCTACGTA-3′	1007 bp	55	[33]
*arp*A	F: 5′-AACGCTATTCGCCAGCTTGC-3′ R: 5′-TCTCCCCATACCGTACGCTA-3′	400 bp	59	[34]
*chu*A	F: 5′-ATGGTACCGGACGAACCAAC-3′ R: 5′-TGCCGCCAGTACCAAAGACA-3′	288 bp	59	[34]
*yja*A	F: 5′-CAAACGTGAAGTGTCAGGAG-3′ R: 5′-AATGCGTTCCTCAACCTGTG-3′	211 bp	59	[34]
TspE4C2	F: 5′-CACTATTCGTAAGGTCATCC-3′ R: 5′-AGTTTATCGCTGCGGGTCGC-3′	152 bp	59	[34]
*trp*A (Group C)	F: 5′-AGTTTTATGCCCAGTGCGAG-3′ R: 5′-TCTGCGCCGGTCACGCCC-3′	219 bp	59	[35]
*arp*A (Group E)	F 5′-GATTCCATCTTGTCAAAATATGCC-3′ R 5′GAAAAGAAAAAGAATTCCCAAGAG-3′	301 bp	57	[35]
*trp*A (Internal control)	F: 5′-CGGCGATAAAGACATCTTCAC-3′ R: 5′-GCAACGCGGCCTGGCGGAAG-3′	489 bp	59	[34]

**Table 2:** Molecular characterization and antimicrobial resistance profile of *bla_CTX-M_*-positive *Escherichia coli* isolates (*n* = 22) derived from chicken meat.

Isolate Number	*bla_SHV_*	*bla_CTX-M_*	*bla_CMY_*	*bla_TEM_*	*bla_OXA_*	Phylogroup	Antibiotic-Resistance Profile
5	ND	+	ND	ND	ND	B1	AMP, ATM, CEZ, CRO, CIP, GM, MIN, LVX, PIP, TET
6	ND	+	ND	ND	ND	B1	AMP, CEZ, CRO, GM, PIP, TET
9	ND	+	ND	ND	ND	B1	AMP, SAM, CEZ, CRO, GM, PIP, TET
10	ND	+	ND	ND	ND	B1	AMP, CEZ, CRO, GM, PIP, TET
13	ND	+	ND	ND	ND	B1	AMP, CEZ, CRO, GM, PIP, TET
14	ND	+	ND	ND	ND	B1	AMP, CEZ, CRO, GM, PIP, TET
16	ND	+	ND	ND	ND	F	AMP, CEZ, CRO, CIP, GM, MIN, LVX, PIP, TET, SXT
17	ND	+	ND	ND	ND	F	AMP, CEZ, CRO, CIP, GM, LVX, PIP, TET, TOB, SXT
38	ND	+	+	ND	ND	A	AMP, SAM, CEZ, CAZ, CRO, CIP, GM, MIN, PIP, TET, SXT
39	ND	+	ND	+	ND	B1	AMP, SAM, ATM, CEZ, CAZ, CRO, GM, MIN, PIP, TET
40	ND	+	ND	+	ND	B1	AMP, SAM, ATM, CEZ, CAZ, CRO, CIP, MIN, PIP, TET
50	ND	+	ND	+	ND	A	AMP, SAM, CEZ, CRO, CIP, GM, MIN, LVX, PIP, TET, SXT
52	ND	+	ND	+	ND	A	AMP, SAM, CEZ, CRO, CIP, GM, LVX, PIP, TET, SXT
53	ND	+	ND	ND	ND	B1	AMP, CEZ, CRO, CIP, TET
65	ND	+	ND	+	ND	A	AMP, SAM, CEZ, CRO, CIP, GM, LVX, PIP, TET, SXT
70	ND	+	ND	+	ND	A	AMP, SAM, CEZ, CRO, CIP, GM, LVX, PIP, TET
71	ND	+	ND	+	ND	F	AMP, SAM, CEZ, CRO, CIP, GM, PIP, TET
73	ND	+	ND	+	ND	B1	AMP, SAM, CEZ, CRO, CIP, GM, LVX, PIP, TET, SXT
80	ND	+	ND	ND	ND	C	AMP, SAM, CEZ, CRO, CIP, PIP, TET, SXT
86	ND	+	ND	ND	ND	C	AMP, ATM, CEZ, CRO, CIP, GM, LVX, PIP, TET, TOB, SXT
87	ND	+	ND	ND	ND	C	AMP, ATM, CEZ, CRO, CIP, GM, LVX, PIP
96	ND	+	ND	ND	ND	B1	AMP, CEZ, CRO, CIP, GM, LVX, PIP, TET, SXT

Legend: AMP: ampicillin (R ≥ 32 μg/mL); SAM: ampicillin-sulbactam (R ≥ 32/16 μg/mL); CEZ: cefazolin (R ≥ 32 μg/mL); CAZ: ceftazidime (R ≥ 16 μg/mL); CRO: ceftriaxone (R ≥ 4 μg/mL); TET: tetracycline (R ≥ 16 μg/mL); GM: gentamycin (R ≥ 16 μg/mL); ATM: aztreonam (R ≥ 16 μg/mL); CIP: ciprofloxacin (R ≥ 4 μg/mL); PIP: piperacillin (R ≥ 128 μg/mL); LVX: levofloxacin (R ≥ 8 μg/mL); TOB: tobramycin (R ≥ 16 μg/mL); SXT: trimethoprim-sulfamethoxazole (R ≥ 4/76 μg/mL); MIN: minocycline (R ≥ 16 μg/mL). The minimum inhibitory concentration (MIC) breakpoints follow the CLSI [28] and EUCAST [29] guidelines. The phylogrouping was detected by the quadruplex phylotyping PCR method according to Clermont et al. [34]. R: resistant. ND: not detected.

**Table 3:** Molecular characterization and antimicrobial resistance profile of *bla_CMY_*-positive *Escherichia coli* isolates (*n* = 9) derived from chicken meat.

Strain Name	*bla_SHV_*	*bla_CTX-M_*	*bla_CMY_*	*bla_TEM_*	*bla_OXA_*	Phylogroup	Antibiotic-Resistance Profile
3	ND	ND	+	+	ND	F	AMP, CEZ, CAZ CRO, TET
4	ND	ND	+	+	ND	A	AMP, SAM, CEZ, CAZ CRO, GM, TET, SXT
7	ND	ND	+	+	ND	F	AMP, SAM, CEZ, CRO, GM, MIN, TET
35	ND	ND	+	ND	ND	C	AMP, CEZ, CRO, CIP, GM, LVX, TET, SXT
37	ND	ND	+	ND	ND	B1	CIP, GM, LVX, TET, TOB
38	ND	+	+	ND	ND	A	AMP, SAM, CEZ, CRO, CIP, GM, PIP, TET, SXT
57	ND	ND	+	+	ND	B1	AMP, TET
63	ND	ND	+	+	ND	B1	AMP, CEZ, TET
67	ND	ND	+	+	ND	F	AMP, CEZ, CRO, TET, SXT

Legend: AMP: ampicillin (R ≥ 32 μg/mL); SAM: ampicillin-sulbactam (R ≥ 32/16 μg/mL); CEZ: cefazolin (R ≥ 32 μg/mL); CAZ: ceftazidime (R ≥ 16 μg/mL); CRO: ceftriaxone (R ≥ 4 μg/mL); TET: tetracycline (R ≥ 16 μg/mL); GM: gentamycin (R ≥ 16 μg/mL); ATM: aztreonam (R ≥ 16 μg/mL); CIP: ciprofloxacin (R ≥ 4 μg/mL); PIP: piperacillin (R ≥ 128 μg/mL); LVX: levofloxacin (R ≥ 8 μg/mL); TOB: tobramycin (R ≥ 16 μg/mL); SXT: trimethoprim-sulfamethoxazole (R ≥ 4/76 μg/mL); MIN: minocycline (R ≥ 16 μg/mL). The minimum inhibitory concentration (MIC) breakpoints follow the CLSI [28] and EUCAST [29] guidelines. The phylogrouping was detected by the quadruplex phylotyping PCR method according to Clermont et al. [34]; R: resistant; ND: not detected.

**Table 4:** Association of B-Lactam resistance genes (*bla_CTX-M_*, *bla_CMY_*, *bla_TEM_*) and phylotypes of *Escherichia coli* isolates illustrated by numbers and (%).

Gene Name	Numbers of the Strains	Phylogroup Numbers and (%)					
		A	B1	C	D	E	F
*bla_CTX-M_*	22	5 (22.72%)	11(50%)	3 (13.63%)	-	-	3 (13.63%)
*bla_CMY_*	9	2 (22.22%)	3 (33.33%)	1 (11.11%)	-	-	3 (33.33%)
*bla_TEM_*	57	22 (38.59%)	24 (42.10%)	2 (3.5%)	3 (5.26%)	2 (3.5%)	4 (7.01%)

## Data Availability

Data is contained within the article or supplementary material.

## Supplementary Materials

The following supporting information can be downloaded at: https://www.mdpi.com/article/10.3390/foods12030598/s1, Figure S1: A representative agarose gel electrophoresis showing the amplified DNA product of the *uidA* gene (the marker gene for *E. coli*) at the expected molecular weight of 1487 bp. Genomic DNA from *E. coli* isolates was used as a template; Figure S2: A representative agarose gel electrophoresis for the PCR-amplified DNA products of the β-lactamase encoding genes *bla_CTX-M_* (A), *bla_CMY_* (B), and *bla_TEM_* (C), at the expected molecular weight of 550, 1007, and 1080 bp, respectively; Figure S3: A representative agarose gel electrophoresis for the PCR-amplified genes incorporated in the phylogroup typing of the confirmed *E. coli* strains. (A): The *trp*A (internal control), *arp*A, *chu*A, and *yja*A were amplified as quadruplex PCR and determined at the expected molecular sizes of 489, 400, 288, and 211 bp, respectively Agarose gel electrophoresis of a singleplex-PCR for the amplification of *trp*A (group C), TspE4C2, and *arp*A (group E) which were verified at the expected molecular size of 219, 152, and 301 pb, respectively.
