# Supplementary material for: β-Lactamase Producing Escherichia coli Encoding blaCTX-M and blaCMY Genes in Chicken Carcasses from Egypt
Source: Foods. 2023 Feb 1;12(3):598. doi: 10.3390/foods12030598 (PMC9914308; doi:10.3390/foods12030598)
Supplement: Supplementary file 1 [file foods-12-00598-s001.zip › foods-2151998-supplementary.pdf]

Supplementary file

Figure legends for the Supplementary figures

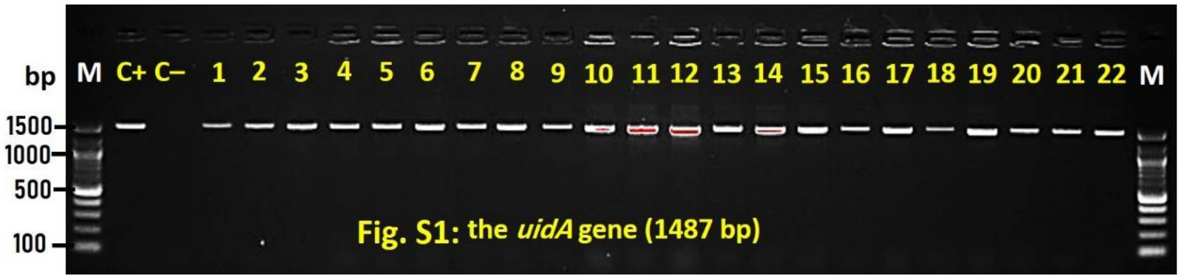

Figure S1: A representative agarose gel electrophoresis showing the amplified DNA product of the *uid*A gene (the marker gene for *E. coli*) at the expected molecular weight of 1487 bp. Genomic DNA from *E.* coli isolates was used as a template.

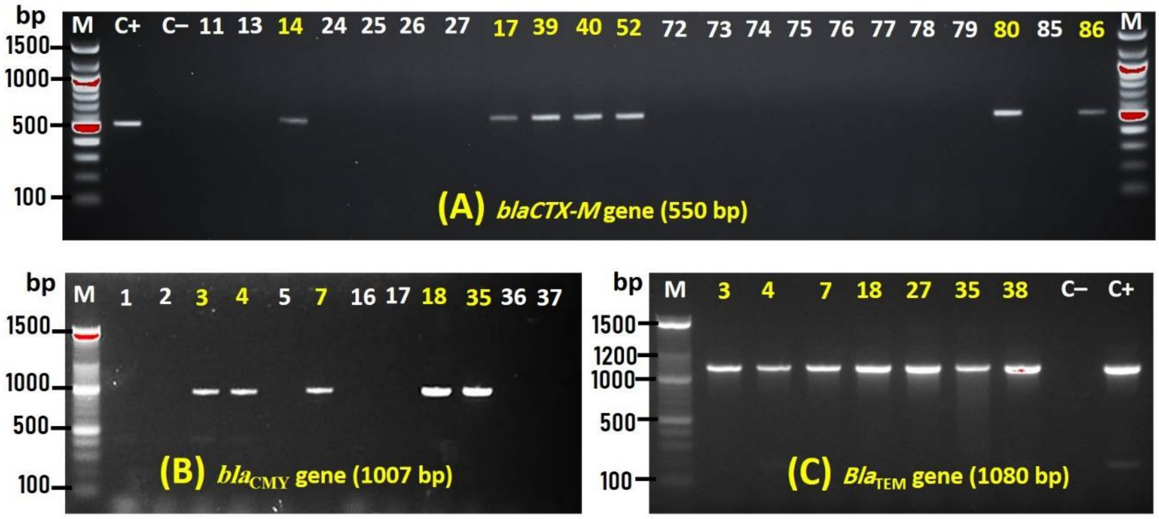

Figure S2: A representative agarose gel electrophoresis for the PCR-amplified DNA products of the β-lactamase encoding genes $bla_{\text{CTX-M}}$ (A), bla_{CMY} (B), and bla_{TEM} (C), at the expected molecular weight of 550, 1007, and 1080 bp, respectively.

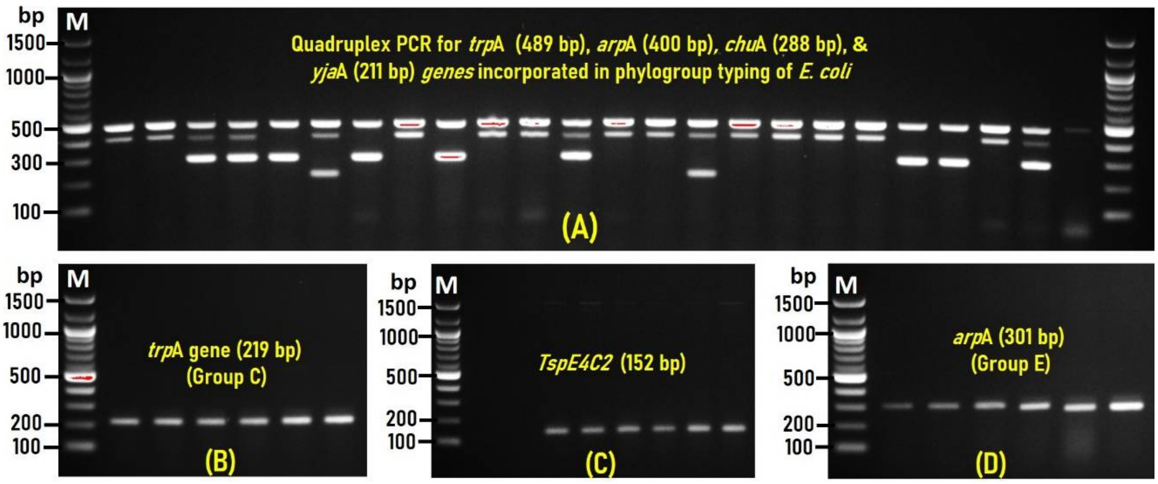

Figure S3: A representative agarose gel electrophoresis for the PCR-amplified genes incorporated in the phylogroup typing of the confirmed *E. coli* strains. (A): The *trp*A (internal control), *arp*A*, chu*A, and *yja*A were amplified as quadruplex PCR and determined at the expected molecular sizes of 489, 400, 288, and 211 bp, respectively Agarose gel electrophoresis of a singleplex-PCR for the amplification of (B) *trp*A (group C), (C) TspE4C2, and (D) *arp*A(group E) which were verified at the expected molecular size of 219, 152, and 301 pb, respectively.
